# Parental morph combination does not influence innate immune function in nestlings of a colour-polymorphic African raptor

**DOI:** 10.1038/s41598-021-90291-7

**Published:** 2021-05-26

**Authors:** Carina Nebel, Arjun Amar, Arne Hegemann, Caroline Isaksson, Petra Sumasgutner

**Affiliations:** 1 FitzPatrick Institute of African Ornithology, DSI-NRF Centre of Excellence, University of Cape Town, Cape Town, South Africa; 2 Department of Biology, Lund University, Lund, Sweden; 3 Konrad Lorenz Research Centre (KLF), Core Facility for Behaviour and Cognition, Department of Behavioral and Cognitive Biology, University of Vienna, Vienna, Austria; 4 Department of Biology, University of Turku, Turku, Finland

**Keywords:** Ecology, Immunology, Zoology

## Abstract

Conditions experienced during early life can have long-term individual consequences by influencing dispersal, survival, recruitment and productivity. Resource allocation during development can have strong carry-over effects onto these key parameters and is directly determined by the quality of parental care. In the black sparrowhawk (*Accipiter melanoleucus*), a colour-polymorphic raptor, parental morphs influence nestling somatic growth and survival, with pairs consisting of different colour morphs (‘mixed-morph pairs’) producing offspring with lower body mass indices, but higher local apparent survival rates. Resource allocation theory could explain this relationship, with nestlings of mixed-morph pairs trading off a more effective innate immune system against somatic growth. We quantified several innate immune parameters of nestlings (hemagglutination, hemolysis, bacteria-killing capacity and haptoglobin concentration) and triggered an immune response by injecting lipopolysaccharides. Although we found that nestlings with lower body mass index had higher local survival rates, we found no support for the proposed hypothesis: neither baseline immune function nor the induced immune response of nestlings was associated with parental morph combination. Our results suggest that these immune parameters are unlikely to be involved in providing a selective advantage for the different colour morphs’ offspring, and thus innate immunity does not appear to be traded off against a greater allocation of resources to somatic growth. Alternative hypotheses explaining the mechanism of a low nestling body mass index leading to subsequent higher local survival could be related to the post-fledgling dependency period or differences in dispersal patterns for the offspring from different morph combinations.

## Introduction

Individual fitness can be determined by conditions experienced during early-life stages. These “silver spoon effects” have been found in a wide range of animal taxa^1,2^ and can impact different demographic parameters throughout life, such as dispersal^3–6^, reproductive performance^4,7–10^, survival^9,10^ and senescence^7,11^. The mechanisms underlying such long lasting effects are less understood, but range from an improved ability to cope with stressors^12^ or competitors^13,14^ to stronger immunocompetence^15^. The variability of such early-life experiences are mainly associated with environmental factors^1,2^ and parental care, which, in turn, might be influenced by parental quality^3,16^, parental phenotype^17–23^ or parents’ experiences^24^.

Nestlings of altricial bird species are completely reliant on the resources provided by their parents^25,26^. When these resources are limited, individuals must allocate them amongst competing functions, such as somatic growth or the development of the immune system^27–29^. These trade-offs will have profound impacts on an individual’s fitness and are thus likely to be under strong selection. Understanding an individual’s development and which specific traits translate into long-term fitness effects is key to unravelling the ecological and evolutionary processes^2^ that can shape an individual’s life history.

In species with biparental care, males and females might have different strategies when allocating resources to their offspring. Likewise, parental investment might also vary between individuals^30–32^ which can result in offspring differing in quality. Within colour-polymorphic species, morphs may display different parental strategies^18,33^. For example, different colour morphs in white-throated sparrows (*Zonotrichia albicollis*) show differential investment in parental care^19^. Thus, the young of different parental morph combinations may be exposed to different early-life experience and this may have carry-over effects on their survival and other fitness related traits.

The black sparrowhawk (*Accipiter melanoleucus*) is a colour-polymorphic raptor that occurs as light or dark morph adults^34^. Although there is no indications of morph-dependant mating patterns^35^, the combination of morphs within a pair (hereafter ‘pair morph’, if consisting of contrasting morphs, termed ‘mixed-morph’ or if consisting of the same morph, termed ‘like-morph’ pairs) have been shown to affect reproductive performance: Mixed-morph pairs have a higher probability of breeding successfully^35^. Offspring from mixed-morph pairs also have been found to be associated with a lower body mass index^35^, but higher subsequent local survival (i.e., nestlings of mixed-morph pairs have a higher chance to remain in the population with higher apparent survival rates)^17^. Although local survival and nestling body mass index have not yet been mechanistically linked in this species, this runs counter to the usual assumption that nestlings that fledge with a lower body mass index will have lower survival or recruitment rates^21,36–41^.

Individual quality can also be assessed by measures other than body mass index, for example by examining parameters linked to the expression of the immune system^27,42,43^. The innate immune system is the first line of defence against pathogens without the need of prior exposure^44^. Due to its importance in maintaining an individual’s health, it plays a crucial role in survival^45–47^, especially in young that are still developing their immune function^29,48,49^. However, immune function is a costly trait to invest in^50,51^, which can thus be associated with negative fitness consequences. For example, individuals might show lower long-term survival after mounting an immune response^52,53^. To quantify the innate immune system of black sparrowhawk nestlings, we use a combined approach; whereby we measure several components of baseline innate immune function and perform an experimental endotoxin immune challenge to examine immune response. The measured baseline innate immune parameters are the complement system (hemolysis, HL), natural antibodie (hemagglutination, HA), bacteria-killing capacity (BK), which play an important role in host defense against infectious agents^54,55^, and haptoglobin (Hp), which is an acute-phase protein^56–58^. Haptoglobin is released by the liver during infections, and its concentration is usually negatively correlated with an individual’s body mass index^59^. For the innate immune challenge, we use lipopolysaccharides (LPS) that naturally occur on the outer surface membrane of bacteria and mimic a bacterial infection when injected^60^. All components of this study focus on the innate immune function, which is better developed than the acquired immune function in young birds^29,48,49^. By combining multiple assays as well as measuring both baseline innate (constitutive) immune function and an experimentally induced innate immune response, which are regulated differently and have different costs, our study aims for a more complete view of the immune system^59,61–63^.

According to alternative resource allocation theory, individuals must allocate resources to competing functions^27,28^. Offspring from mixed-morph pairs might trade-off investment in immune function at the expense of somatic growth, whereas nestlings of like-morph pairs might invest more in somatic growth and less in immune function and this may explain why mixed-morph offspring have a lower average body mass index. This ‘alternative resource allocation’ hypothesis could also explain why mixed-morph offspring have better local survival rates as their higher investment in baseline immune function could provide them with a selective advantage against pathogens^64,65^ and higher baseline immune function is often associated with higher survival^45–47^. At the same time, investment in baseline immune function might reduce the risk that pathogens start establishing themselves in the body which requires an innate immune response. Such immune responses can have negative physiological effects such as immunopathology^66^ and can result in short- and long-term reduction of fitness^50,52,53,67^). Differences in resource allocation could be caused by parental morph-dependant prey delivery behaviour. Whilst black sparrowhawk pair morph does not influence overall amount of prey delivered to nestlings, it can influence consistency in food delivery, with more regular feeding patterns observed for mixed-morph pairs^20^. These differences may promote differential physical and physiological investment between the offspring of mixed- and like-morph pairs.

In this study, we use three datasets to explore alternative resource allocation between somatic growth, innate immune function and local survival by offspring, by using mark-recapture (‘apparent local survival’) data from individuals encountered between 2001 and 2019, correlative innate immune data collected between 2015 and 2019 and experimentally challenged immune response data collected in 2018 and 2019. Due to their elusive nature during non-breeding, survival of black sparrowhawks is usually assessed with recruitment into the breeding population which happens at approximately 2.8 years of age^17^. As a result, we assess nestling innate immune function and subsequent survival rates indirectly. Under the ‘alternative resource allocation’ hypothesis, we would predict that (i) immune function of nestlings of mixed-morph pairs is higher than those of like-morph pairs, (ii) birds with higher immune function will have a leaner body (low body mass index); and (iii) nestlings with a lean body have higher apparent survival rates. In more detail, we predict nestlings of mixed-morph pairs (known to have a lower average body mass index^35^) to exhibit a stronger complement system, natural antibodies and bacteria-killing capacity than like-morph chicks, because we predict they allocate more resources into their immunological development than somatic growth. Furthermore, we predict that nestlings of mixed-morph pairs show lower haptoglobin concentrations because the acute phase protein haptoglobin usually increases during inflammation and we expect nestlings of mixed-morph pairs to be healthier than those of like-morph pairs. However, hemolytic anemia (i.e. caused by malaria infection) can result in decreased haptoglobin levels in humans^68^, thus, the reverse relationship is also possible, with weaker birds having lower haptoglobin levels. Such a result would indicate that nestlings of mixed-morph pairs invest more into the development of innate immune function and are thus able to fight pathogens more effectively, which should result in low infection and inflammation rates. Lastly, we predict nestlings of mixed-morph pairs to show a stronger response to the mimicked bacterial infection, which under attack by a real pathogen would mean a faster clearance of the pathogen.

## Results

### Baseline immune function in relation to pair morph

**Figure 1:**
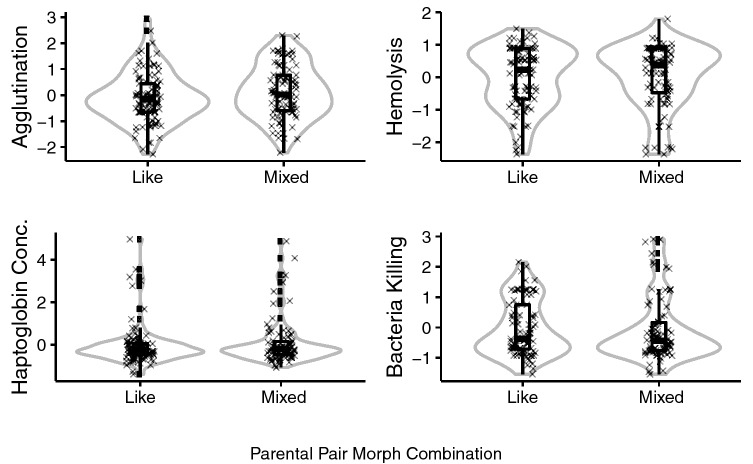
Baseline innate immune parameters (scaled values and centred to the mean of the year) in relation to pair morph combination (‘like-morph’: dark-dark D♂D♀ and light-light L♂L♀; or ‘mixed-morph’: dark–light D♂L♀ and light–dark L♂D♀). Boxplots are based on standardized raw data (individual data values shown as cross marks), outliers shown as black points, grey outlined violin plot gives an estimate of the distribution of data. Differences were not significant.

We found no difference in any of the baseline innate immune parameters in relation to parental pair morph combination (Table 1, Fig. 1, see supplementary table S1–S8 for full model outputs). Variation in baseline innate immune function was, however, associated with some of our other covariates: Female nestlings had a significantly stronger bacteria-killing capacity than male nestlings (estimate = − 0.064, SE = 0.024, N_df=1,161_ = 168, χ^2^ = 7.94, P = 0.007, table S7, table S8). Haptoglobin levels varied with season, with nestlings hatched earlier having higher haptoglobin levels than later hatched chicks (estimate = − 0.022, SE = 0.011, N_df=1,169_ = 177, χ^2^ = 4.03, P = 0.045, table S5, table S6). Haptoglobin levels also varied with brood size, with nestlings in larger broods having higher haptoglobin concentrations (N_df=1,169_ = 177, χ^2^ = 4.02, P = 0.045; Hp concentration of single chick broods = 0.178 (SE = 0.063), broods with two chicks = 0.147 (SE = 0.035), broods with three chicks = 0.125 (SE = 0.016). Note that the discrepancy with broods with three chicks showing the lowest level in the raw data is due to three extreme points. The negative relationship is turned into a positive after log-transforming the haptoglobin concentration to meet the requirement of normally distributed residuals in a linear mixed model.

**Table 1:** Model outputs of linear mixed models for the key explanatory variables of the ‘baseline innate immune models’ (response variables: hemagglutination, hemolysis, bacteria-killing capacity and baseline haptoglobin) and ‘innate immune response model’ (response variable: haptoglobin response, quantified as the difference between baseline haptoglobin concentration and haptoglobin concentration post-injection of lipopolysaccharides).

Measurement	Study period	N		Model output ‘pair morph’ explanatory variable						Model output ‘body mass index’ explanatory variable					
		Like	Mixed	Estimate	SE	χ2	ndf	ddf	P	Estimate	SE	χ2	ndf	ddf	P
**Baseline innate immune models**															
Hemagglutination	2015–2019	98	81	0.247	0.172	2.07	1	172	0.156	**0.003**	**0.002**	**3.94**	**1**	**172**	**0.047**
Hemolysis	2015–2019	98	81	0.030	0.147	0.04	1	172	0.837	0.002	0.001	1.34	1	172	0.225
Baseline haptoglobin	2015–2019	99	78	0.012	0.021	0.32	1	169	0.574	-0.001	0.001	0.24	1	169	0.624
Bacteria killing	2015–2019	91	77	0.008	0.030	0.08	1	161	0.783	0.001	0.001	0.90	1	161	0.344
**Innate immune response model**															
Haptoglobin response	2018–2019	30	19	-0.158	0.307	0.27	1	41	0.607	-0.001	0.003	0.16	1	41	0.694

Key explanatory variables are parental ‘pair morph’ (factor in two levels: ‘like-morph’: dark-dark D♂D♀ and light-light L♂L♀; or ‘mixed-morph’: dark–light D♂L♀ and light–dark L♂D♀, the reference category is ‘mixed-morph') or the ‘body mass index’. All ‘baseline innate immune models’ were fitted with individual ‘sex’ (male or female), ‘age’ (numeric, in days: 20–35), ‘brood size’ (numeric, 1–3), ‘seasonality’ (numeric, week number: 22–46), and ‘time of the day’ when sample was obtained (numeric, hour: 7–17) as additional co-variates and territory ID as a random factor. Bacteria-killing and baseline haptoglobin concentration were log-transformed, the baseline haptoglobin model included an additional co-variate: a reading at 450 nm to control for plasma redness. The ‘innate immune response model’ was fitted with two baseline readings (650 nm and 450 nm) to control for initial haptoglobin concentration and plasma redness, ‘sex’, ‘age’ (numeric, week number: 22–35), ‘brood size’, ‘seasonality’ (numeric, week number: 23–43), as co-variates and territory ID as a random factor. All response variables were scaled and centred to the mean of the year, whereas all continuous variables were scaled. Study period (in years), sample size (N, for mixed- and like-morph), numerator and denominatior degrees of freedom (ndf and ddf) given in respective columns. See tables S1-S9 for full model outputs.

### Innate immune response in relation to pair morph

**Figure 2:**
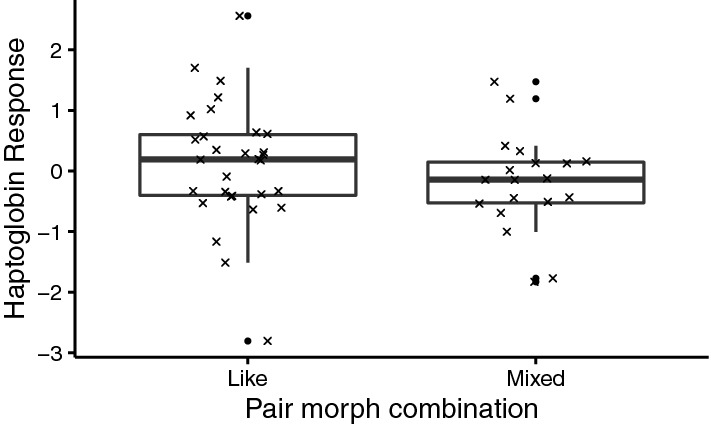
Haptoglobin response (scaled values and centred to the mean of the year) in relation to pair morph combination (‘like-morph’: dark-dark D♂D♀ and light-light L♂L♀; or ‘mixed-morph’: dark–light D♂L♀ and light–dark L♂D♀). The haptoglobin response variable is the difference in haptoglobin concentration between the baseline haptoglobin and the concentration measured after the injection of lipopolysaccharides (LPS). Cross marks are scaled raw values, black dots outliers. All values scaled and centred to the mean of the collection year to remove between-year variation. Differences were not significant.

The post-exposure haptoglobin levels of nestlings injected with LPS were higher than their baseline pre-exposure the haptoglobin levels (estimate: − 0.912, SE = 0.166 SE, N_df=1,46_ = 49, χ^2^ = 23.71, P < 0.001; baseline Hp conc.: 0.28 (SD = 0.48), post-exposure Hp conc.: 0.63 (SD = 0.50), however, the haptoglobin response did not differ between pair morphs (χ^2^ = 0.27, P = 0.610, Fig. 2), nor with any of our other covariates (see supplementary table S9 and S10 for full model outputs).

### Relationship between nestling body mass index and immune function

**Figure 3:**
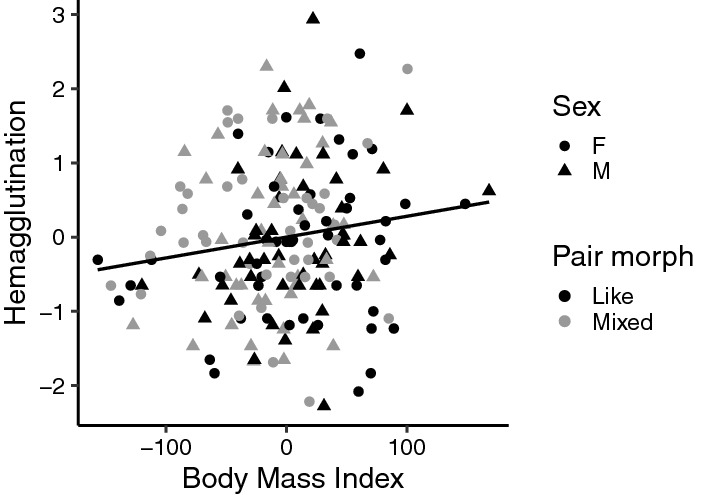
Relationship between hemagglutination titre score and body mass index. Each symbol represents one observation (nestling). Colour represent parental pair morph combination (pair morph): black symbols are offspring produced by like-morphs, grey symbols offspring of mixed-morph parents. Circles are female nestlings, triangles males. Trend line is the linear regression of hemagglutination and the body mass index (y = 0.002x + 0.006, R^2^ = 0.019). The hemagglutination titre values were scaled and centred to the mean of the year.

We found no negative relationship between any baseline innate immune parameter or the innate immune response in relation to the nestling body mass index (Table 1). Instead, for the hemagglutination titre, we found a marginally significant positive association with body mass index, albeit effect sizes were small (estimate = 0.003, SE = 0.002, N_df=1,172_ = 179, χ^2^ = 3.94, P = 0.047, Fig. 3); indicating higher hemagglutination levels for nestlings in better condition.

### Apparent local survival in relation to body mass index

**Figure 4:**
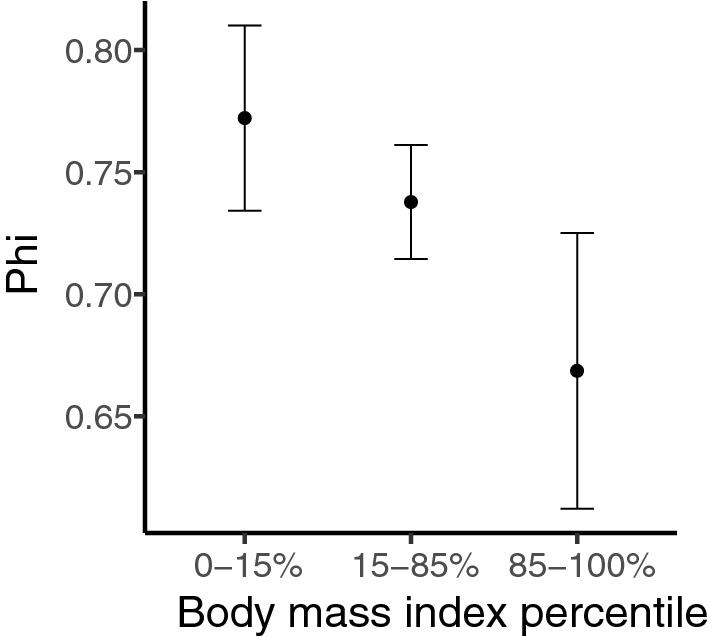
Apparent survival (Φ) estimates and standard error of nestling black sparrowhawks in relation to body condition percentile groups (group 1, 0–15%: 0.77 ± 0.04 SE; group 2, 15–50%: 0.74 ± 0.02 SE; group 3, 85–100%: 0.67 ± 0.06 SE). Re-sightings data was collected between 2001 and 2019 (N = 438).

Although apparent survival (Φ) displayed a negative relationship with increasing body mass index (Fig. 4), variation around these estimates were relatively large, and our CONTRAST analysis indicated that the difference between these groups were not statistically significant; contrast between lean and heavy nestlings, χ^2^ = 1.27, N = 132, P = 0.260) after correcting for overdispersion.

## Discussion

We proposed an alternative resource allocation hypothesis to explain why black sparrowhawk offspring from mixed morph parents produced nestlings with a low body mass index but with higher apparent survival rates. We found no support for our first prediction, that innate immune function of nestlings of mixed-morph pairs is higher than those of like-morph pairs. Furthermore, we found no support of our second prediction, that birds with higher immune function will have a leaner body (i.e. low body mass index); only one immune parameter was associated with nestling body mass index, and this relationship ran counter to our predictions, with higher hemagglutination levels for nestlings with a heavier body (i.e. higher body mass index). Our third prediction was that nestlings with a lean body would have higher apparent survival rates. Although there was an indication for higher local survival rates for nestlings with the lowest body mass index compared to nestlings with the highest body mass index, these differences were not significant.

Contrary to our prediction, black sparrowhawks in good physical condition also showed the best natural antibody titre (hemagglutination). In general, this finding is in line with observations in other species showing associations between a high body mass index and strong immune-competence^38,69,70^. However, in the present study system, in which nestlings with a low body mass index show the highest local survival rates, the association of a high body mass index and high innate immune function challenges the original idea that a strong innate immune system is linked to higher local survival rates. Natural antibodies do not need prior exposure to antigens and play an important role in facilitating pathogen recognition and clearance of apoptotic cells^71–73^, especially in young that still do not have a well-developed acquired immune system^72^. If resources are limited, animals are known to redistribute their investment from energetically expensive immune functions to cheaper ones, like natural antibodies^54,63,74,75^. This could be the case for black sparrowhawks, whereby nestlings that invest more into somatic growth, may down-regulate expensive and upregulate cheap immune parameters. However, we did not find any indication of a down-regulation of expensive innate immune function, i.e. bacteria-killing capacity and complement system^54,63^, which does not provide support for this hypothesis of redistribution among innate immune functions. Due to the limited number of resightings of the nestlings for which we have data of immune measurements, we currently cannot fully disentangle the relationship of somatic growth, natural antibodies and survival.

The apparent lack of a trade-off between innate immunological and physical development could be due to multiple reasons: First, individual variation can make it difficult to unravel trade-offs, especially when working with field data^76,77^. Second, the innate immune system is complex and consists of many components and physiological relationships that can interact with one another. Although we used multiple parameters to gain a more complete picture, our chosen parameters might not be suitable to reveal differences in resource allocation. Third, differences in the innate immune function might only become visible in a later development stage, i.e. during the post-fledging dependency period. Fourth, differences might be only prevalent in the acquired immune system.

Another explanation for the higher apparent survival of mixed morph pairs, may be linked to emigration. Sumasgutner et al. (2016) found that offspring of mixed-morph pairs had higher apparent survival, but such survival estimates may be determined by a combination of survival and emigration out of the study area^78^. Thus, higher apparent survival for chicks produced by mixed-morph pairs could also emerge if offspring from like-morph pairs were more likely to disperse or if dispersal was positively associated with the body mass index. Support for a body condition-dependant juvenile dispersal in raptors can be found in Northern goshawks (*Accipiter gentilis*)^79^ and Eurasian kestrels (*Falco tinnunculus*)^80^. However, such a pattern is not present in all hawks^81,82^. Unfortunately, with only a handful of re-sightings of colour-ringed individuals from outside our intensely monitored study population, exploring this possibility is unfeasible at present.

Our study has revealed sex-specific differences in the innate immune system with female nestlings showing stronger bacteria-killing capacities. Such sex-specific differences have previously been reported in other species, and have been linked to sex hormones^83,84^. These relationships might also explain why male animals are prone to larger parasite loads than females^85–87^. In support of this, adult male black sparrowhawks show higher *Haemoproteus* blood parasite infection intensity than adult females^64^, suggesting that sex-specific differences of nestling immune function might continue into adulthood.

Nestlings of broods with three chicks had high levels of haptoglobin concentration, indicating increased levels of inflammation. This might conform with the ‘tasty chick’ hypothesis^38,88^ which posits that one sibling (usually the weakest) might be an increased target for parasites and thus show elevated levels of inflammation. Such a pattern is linked to hatching asynchrony, which also occurs in black sparrowhawks, where nestlings usually hatching at one to three day intervals^89,90^. However, it is difficult to determine hatching rank based on size and plumage development in black sparrowhawks, which shows strong sexual size dimorphism, thus such a hypothesis is difficult to test without individual marking at hatching.

The acute-phase protein haptoglobin showed a seasonal effect: Nestlings that were ringed at the beginning of the season showed higher concentrations which declined during the course of the season. This seasonal pattern might be due to higher pathogen pressure during the colder and wetter period at the beginning of the breeding season^64,65,91^, even though early months are characterized by higher breeding success in the population on the Cape peninsula^35,92^. High haptoglobin concentrations during the wet season with potentially higher pathogen pressure may indicate that nestlings rely on high baseline values which enable a faster response to infection^93,94^. High breeding success during the early months of the breeding season could then be a combination of earlier breeders being of higher individual quality and/or occupying higher quality territories (e.g., “sequential settlement”^95^. Low haptoglobin concentrations towards the end of the breeding season could accordingly reflect lower pathogen pressure^93,94^.

## Conclusion

The previously found low body mass index of nestlings of mixed-morph pairs, but higher apparent survival rates of these offspring suggests that nestlings are following different investment strategies and trade off physical development for other aspects of growth, i.e. the innate immune system. However, our study did not reveal such an association. Nestlings of mixed-morph pairs showed the same baseline innate immune levels and innate immune response as compared to nestlings of like-morph pairs. Although the innate immune system is usually a good predictor of survival, our findings imply that differences in the innate immune system are unrelated to differences in survival in our study system. Furthermore, contradicting to our prediction, nestlings with a high body mass index also showed the highest natural antibody titre. This finding indicates potential contrasts in up- and downregulation of different immune parameters, although we found no evidence of such a redistribution taking place. While we did not find support for any of our predictions, our study is the first to present data on the innate immune system of nestlings of a colour-polymorphic *Accipiter* hawk. Future studies should focus on the post-fledgling and dispersal behaviour of black sparrowhawk juveniles which might be shaped by parental morphs and could explain the observed life-history traits.

## Material and methods

### Study system and productivity data

The black sparrowhawk study population is located on the Cape peninsula (34°00′ S, 18°26′ E), Western Cape, South Africa. Each year, 11 to 45 (mean 32) black sparrowhawk territories show breeding activity. Territories were checked from April until October, initially monthly, until breeding activity was recorded and from then on weekly. During visits, the sex and morphs of the territorial pair were identified. Sexes can be easily distinguished in the field with males being considerably smaller than females^89,90^.

### Nestling ringing and body condition measurement

Black sparrowhawk nestlings were fitted with a metal ring supplied by the South African Bird Ringing Unit (SAFRING^96,97^) and a unique colour ring combination at the age of 20 to 35 days. Systematic ringing of black sparrowhawk nestlings started in 2006, but four individuals were ringed at nestling age in 2001 and 2003 and were included in our survival analysis. Subsequent re-sightings of these marked birds were recorded annually between 2001 and 2019 and allowed survival estimates to be obtained. Nestlings were aged by comparing their plumage development with reference photos of chicks of known age^98^. During ringing, nestlings were weighted using a scale (to the nearest 1 g) and their tarsus length was measured using a calliper (to the nearest 0.1 mm). To derive the body mass index, we extracted the residuals of a linear regression between body mass and tarsus length, controlling for sex.

### Blood samples and immune challenge

When nestlings were ringed, we also took a blood sample. Blood samples were taken within 20 min of removing them from the nest, well within the time period where no handling effect is expected on the nestling’s immune function^99,100^. A blood sample was taken from the brachial vein for the baseline immune analysis, from 2015 to 2019. In 2018 and 2019, an immune challenge was performed in addition to the baseline sampling. At these nests, after taking the first blood sample for the baseline immune analysis, the immune challenge was carried out: nestlings were injected with a 1 mg/kg LPS solution (in phosphate-buffered saline, PBS) subcutaneously on the breast. A second blood sample (‘post-exposure’) to quantify the response to the endotoxin was taken between 16 to 18 h (mean = 17.17 h, SD = 0.48 h) after the immune challenge. The time window was based on previous studies^101^ as well as logistical constraints. This post-exposure sample was used to determine the change in haptoglobin concentration from the baseline value (see details further below). The field procedure in 2018 and 2019 was always the same: nestlings were injected with LPS in the afternoon (between 15:00 and 17:33), immediately returned to their nest and a second blood sample was taken the next morning (between 08:26 and 11:11). The total blood volume taken per nestling was max. 1 ml in total (approximately 500 µl per bleeding). Directly after blood collection, red blood cells and plasma were separated by centrifuging in the field (10,000 rpm/20 min) and frozen in liquid nitrogen immediately afterwards. All samples were stored at − 80 °C until processing and randomized before laboratory work began.

### Innate immune assay protocols and lab procedures

We measured three different components of the baseline innate immune system. First, the complement system and natural antibodies were assessed via the hemolysis (HL) and hemagglutination (HA) assay^54^. They were determined as the lysis and agglutination titre of 12.5 µl foreign red blood cells (rabbit blood) in a serial dilution of 12.5 µl black sparrowhawk plasma in 12.5 µl PBS. Titre scoring was performed visually after 20 min (hemagglutination) and 90 min (hemolysis) of incubation in a 37 °C distilled water bath and wells counted that showed signs of hemagglutination or hemolysis. Titres were scored twice, both times randomized and blind to sample identity. In case the difference between both scores was larger than one (measured in wells), the titre was scored a third time. The mean of two scores or median of three was then used for the statistical analysis. On each plate, the serial dilution of two chicken (*Gallus gallus domesticus*) plasma samples was used as a control and produced an inter-assay coefficient of variance (CV) of 16.3% (HA) and 10.6% (HL).

Second, the bacteria-killing (BK) assay was carried out following the methods of^102^, but using 2/3rd of the reagents and measuring final bacteria growth at 600 nm^103^. The initial bacteria concentration was 10^5^ *Escherichia coli*/ml and bacteria volume 3.5 µl per well. Plasma volume was 4.5 µl and 8 µl PBS per well. On each plate, a positive (not containing any plasma) and a negative control (not containing any *E. coli* or plasma) were run in quadruplicates. Before incubation, background absorption was measured at 600 nm. Samples were incubated for 12 h at 37 °C before the final absorption reading at 600 nm was done. The bacteria-killing capacity was quantified as the bacteria growth in plasma after 12 h (in %) subtracted by the background absorption in relation to a bacteria positive control that grew on the same plate. Samples were run in triplicates with an intra-assay coefficient of 7.92%.

Third, we measured the haptoglobin concentration of plasma: the baseline concentration and the post-exposure concentration to assess the response of the innate immune system to the LPS injection. We used a commercially available colorimetric assay kit (TP801; Tri-Delta Diagnostics, Maynooth, County Kildare, Ireland) following the manufacturer’s instructions. Haptoglobin concentration was measured at a wavelength of 650 nm. We did an additional reading at 450 nm that was performed directly before adding the final reagent, allowing us to control for plasma redness^57^. A standard provided by the manufacturer was added on every plate and produced an inter-assay CV of 5.44%.

The sample sizes varied between assays because the amount of plasma varied between individuals (Table 1). All innate immune parameters (‘hemolysis’, ‘hemagglutination’, ‘bacteria-killing’, ‘baseline haptoglobin’, ‘haptoglobin response’) and the co-variate ‘plasma redness’ (450 nm haptoglobin measurement) were standardized per year by using the scale function of ‘base’ R, which allowed us to remove any between-year variation that might be caused by differences during transport (samples from 2016 and 2017 thawed during the shipment to the lab, but see^104^), time in storage (2016 samples were stored a year longer before being analysed than samples from other years^104^) or processing. Lab work was done in four batches corresponding to collection years: batch 1 (2015), batch 2 (2016, 2017), batch 3 (2018), batch 4 (2019).

### Statistical analysis

We used R version 3.5^105^ to fit linear mixed models (LMM) using the ‘lmerTest’ package^106^. First, we explored relationships between innate immune function and pair morph , which was fitted as a two-level factor (mixed- or like-morph). The ‘baseline immune’ response variables analysed were ‘hemolysis’, ‘hemagglutination’, ‘bacteria-killing’ and ‘baseline haptoglobin’. Multiple covariates were added to control for their potential influence on baseline innate immune function: nestling’s ‘sex’ (factor variable), to account for sex-specific variation in immune function^107^ and ‘age of the nestlings’ (continuous variable: 20–35, in days), to control for the transitional changes of immune function during development. A nest-specific covariate of ‘brood size’ (continuous variable: 1–3) was included, which controlled for variation in sibling competition experienced^108^. As breeding performance in this species is influenced by timing of breeding^35,92^, we included the calendar week when ringing occurred (1 being the first week of the year, continuous variable: 22–46). Lastly, we controlled for ‘time of the day’ (continuous variable: 7–17, in hours) to account for diurnal patterns in immune function^109^. The bacteria-killing score and baseline haptoglobin concentration were log-transformed to improve normality. For the baseline haptoglobin model we fitted a reading at 450 nm wavelength to control for the redness of the plasma. In all the ‘baseline immune’ models, territory ID was included as a random term. In the ‘hemolysis’ model, the territory ID explained close to 0% of the variance ant thus, its effect is almost negligible (the among-sibling variance in hemolysis is estimated close to zero).

We explored the change of haptoglobin concentration after the injection of LPS. Within this ‘innate immune response’ linear model, we fitted the ‘haptoglobin response’ (post-haptoglobin subtracted by the baseline concentration) as the response variable. We controlled for ‘sex’ (factor variable), ‘age’ of the nestling (continuous variable: 20–32, in days), ‘brood size’ (continuous variable: 1–3), and ‘seasonality’ (continuous variable, week number of ringing: 23–43). In addition, we included the haptoglobin concentration reading (650 nm) from the baseline sample to control for the initial haptoglobin concentration and a reading of the baseline sample at 450 nm to control for ‘plasma redness’^57^. Territory ID was added as a random term to account for the non-independency of siblings.

Linked to the predictions from our alternative resource allocation hypotheses, we directly explored whether there was an association between somatic development (body mass index) and innate immune function, irrespective of parental morph combination. These analyses used the same models as previously described (both the three ‘baseline innate immune’ models and the ‘innate immune response’ model), however, we replaced the ‘pair morph’ explanatory variable with our continuous ‘body mass index’ measure.

All continuous covariates that were used in LMMs, were scaled beforehand (standardized to mean = 0 and SD = 1) in order to bring the variables to comparable dimensions to facilitate interpretation of effect sizes.

### Apparent annual survival in relation to body mass index

We used the packages RMark^110^ and software MARK^111^ to estimate means (and standard errors) of apparent survival (Φ) and re-sighting probability (ρ) of individuals that were ringed as nestlings. We used Cormack-Jolly-Seber (CJS) models with body mass index as a covariate. For this analysis, birds were placed into three groups according to their body mass index, with group 1 (‘lean’) being up to the 15th percentile (from − 153.35 to − 54.12, N = 66), group 2 (‘normal’) between the 15th and 85th percentile (from − 54.12 to 51.59, N = 306) and group 3 (‘heavy’) being above the 85th percentile (51.59–195.12, N = 66). Using the 15% and 85% percentile represents an accurate representation of the body mass index data structure (Figure S1). A goodness-of-fit test using RELEASE (test2 & test3^112^) programs was performed to ensure that data met the homogeneity assumption: χ^2^_df=73_ = 133.411; ĉ = 1.82. Due to the goodness-of-fit indicating overdispersion, standard errors were corrected for ĉ^113^. Post-hoc comparison based on estimates and SEs from extracted coefficients of survival were performed using the CONTRAST package^114^.

### Ethical statement

This study was conducted under CapeNature (Permit no. 0056-AAA041-00099, 0056-AAA007-00105, CN44-30-4175) and SanPark Permits (CRC/2015/009—2012, CRC/2017-18/009—2012/V2) and was approved by the UCT’s ethics committee SFAEC (Permit numbers: 2012/V37/AA, 2016/v11/AA, 2018/v5/AA). We confirm that all methods were carried out in accordance with relevant guidelines and regulations and that the study was carried out in compliance with the ARRIVE guidelines where applicable.

## Supplementary Information

Supplementary Information.

## Data Availability

The dataset can be accessed on Zivahub ^115^.
