# Supplementary material for: Parental morph combination does not influence innate immune function in nestlings of a colour-polymorphic African raptor
Source: Sci Rep. 2021 May 26;11:11053. doi: 10.1038/s41598-021-90291-7 (PMC8155141; doi:10.1038/s41598-021-90291-7)
Supplement: Supplementary file 1 — Supplementary Information. [file 41598_2021_90291_MOESM1_ESM.docx]

SUPPLEMENTARY MATERIAL

**Parental morph combination does not influence innate immune function in nestlings of a colour-polymorphic African raptor**

Carina Nebel*, Arjun Amar, Arne Hegemann, Caroline Isaksson, Petra Sumasgutner

* corresponding author: [carina.nebel@gmail.com](mailto:carina.nebel@gmail.com)

**Full model output hemolysis:**

Table S1 Model output of a linear model exploring the hemolysis score (standardized per year) in relation to parental pair morph combination (either mixed-morph or like-morph, mixed morph as the reference here). Covariates fitted in the model were nestling age (in weeks), brood size, sex (male as reference), season (in weeks) and time of the day. Random effect (territory ID) was removed from the original linear mixed model as it explained 0% of the variance. All continuous variables were scaled. Sample size is 179.

| **Variable** | **Estimate** | **SE** | **χ2** | **ndf** | **ddf** | **P** |
| --- | --- | --- | --- | --- | --- | --- |
| Pair morph | 0.030 | 0.147 | 0.039 | 1 | 172 | 0.837 |
| Nestling age (in days) | 0.018 | 0.073 | 0.057 | 1 |  | 0.802 |
| Brood size | 0.019 | 0.071 | 0.064 | 1 |  | 0.792 |
| Sex | -0.081 | 0.143 | 0.285 | 1 |  | 0.577 |
| Season (week number) | 0.021 | 0.076 | 0.071 | 1 |  | 0.781 |
| Time of the day (hour) | 0.038 | 0.073 | 0.247 | 1 |  | 0.603 |
| Intercept | 0.043 | 0.121 | 0.114 | 1 |  | 0.724 |

Table S2 Model output of a linear mixed model exploring the hemolysis score (standardized per year) in relation to individual body condition. Covariates fitted in the model were nestling age (in weeks), brood size, sex (male as reference), season (in weeks) and time of the day. Radom effect (territory ID) was removed from the original linear mixed model as it explained 0% of the variance. All continuous variables were scaled. Sample size is 179.

| **Variable** | **Estimate** | **SE** | **χ2** | **ndf** | **ddf** | **P** |
| --- | --- | --- | --- | --- | --- | --- |
| Body mass index | 0.002 | 0.001 | 1.340 | 1 | 172 | 0.225 |
| Nestling age (in days) | -0.003 | 0.075 | 0.001 | 1 |  | 0.969 |
| Brood size | 0.030 | 0.071 | 0.160 | 1 |  | 0.674 |
| Sex | -0.090 | 0.145 | 0.346 | 1 |  | 0.537 |
| Season (week number) | 0.021 | 0.074 | 0.070 | 1 |  | 0.780 |
| Time of the day (hour) | 0.029 | 0.073 | 0.140 | 1 |  | 0.694 |
| Intercept | 0.053 | 0.106 | 0.362 | 1 |  | 0.548 |

**Full model output hemagglutination:**

Table S3 Model output of a linear mixed model exploring the hemagglutination score (standardized per year) in relation to parental pair morph combination (either mixed-morph or like-morph, mixed morph as the reference here). Covariates fitted in the model were nestling age (in weeks), brood size, sex (male as reference), season (in weeks) and time of the day. Random effect was territory ID. All continuous variables were scaled. Sample size is 179.

| **Variable** | **Estimate** | **SE** | **χ2** | **ndf** | **ddf** | **P** |
| --- | --- | --- | --- | --- | --- | --- |
| Pair morph | 0.247 | 0.172 | 2.066 | 1 | 172 | 0.156 |
| Nestling age (in days) | -0.055 | 0.077 | 0.502 | 1 |  | 0.480 |
| Brood size | 0.002 | 0.008 | 0.001 | 1 |  | 0.983 |
| Sex | 0.001 | 0.151 | 0.000 | 1 |  | 0.996 |
| Season (week number) | 0.004 | 0.084 | 0.023 | 1 |  | 0.961 |
| Time of the day (hour) | 0.080 | 0.077 | 1.054 | 1 |  | 0.306 |
| Intercept | -0.139 | 0.139 | 0.672 | 1 |  | 0.416 |

Table S4 Model output of a linear mixed model exploring the hemagglutination score (standardized per year) in relation to individual body condition. Covariates fitted in the model were nestling age (in weeks), brood size, sex (male as reference), season (in weeks) and time of the day. Random effect was territory ID. All continuous variables were scaled. Statistically significant variable is indicated in bold. Sample size is 179.

| **Variable** | **Estimate** | **SE** | **χ2** | **ndf** | **ddf** | **P** |
| --- | --- | --- | --- | --- | --- | --- |
| Body mass index | **0.003** | **0.002** | **3.937** | **1** | **172** | **0.047** |
| Nestling age (in days) | -0.087 | 0.079 | 1.211 | 1 |  | 0.271 |
| Brood size | 0.019 | 0.078 | 0.057 | 1 |  | 0.811 |
| Sex | -0.023 | 0.151 | 0.023 | 1 |  | 0.879 |
| Season (week number) | -0.011 | 0.083 | 0.017 | 1 |  | 0.898 |
| Time of the day (hour) | 0.061 | 0.078 | 0.624 | 1 |  | 0.430 |
| Intercept | 0.011 | 0.114 | 0.009 | 1 |  | 0.925 |

**Full model output baseline haptoglobin:**

Table S5 Model output of a linear mixed model exploring the baseline haptoglobin concentration (standardized per year) in relation to parental pair morph combination (either mixed- or like-morph, mixed morph as the reference here). Covariates fitted in the model were nestling age (in weeks), brood size, sex (male as reference), season (in weeks), time of the day (in hours) and a reading at 450 nm to control for plasma redness. Random effect was territory ID. The response variable haptoglobin was log-transformed, all continuous variables were scaled. Statistically significant variables indicated in bold. Sample size is 177.

| **Variable** | **Estimate** | **SE** | **χ2** | **ndf** | **ddf** | **P** |
| --- | --- | --- | --- | --- | --- | --- |
| Pair morph | 0.012 | 0.021 | 0.320 | 1 | 169 | 0.574 |
| Nestling age (in days) | 0.008 | 0.010 | 0.524 | 1 |  | 0.470 |
| **Brood size** | **0.020** | **0.010** | **4.023** | **1** |  | **0.047** |
| Sex | -0.032 | 0.020 | 2.516 | 1 |  | 0.115 |
| **Season (week number)** | **-0.022** | **0.011** | **4.030** | **1** |  | **0.047** |
| Time of the day (hour) | 0.017 | 0.010 | 2.697 | 1 |  | 0.102 |
| 450nm | -0.006 | 0.013 | 0.201 | 1 |  | 0.654 |
| **Intercept** | **0.372** | **0.017** | **457.041** | **1** |  | **<0.001** |

Table S6 Model output of a linear mixed model exploring the baseline haptoglobin concentration (standardized per year) in relation to individual body condition. Covariates fitted in the model were nestling age (in weeks), brood size, sex (male as reference), season (in weeks), time of the day (in hours) and a reading at 450 nm to control for plasma redness. Random effect was territory ID. The response variable haptoglobin was log-transformed, all continuous variables were scaled. Statistically significant variables indicated in bold. Sample size is 177.

| **Variable** | **Estimate** | **SE** | **χ2** | **ndf** | **ddf** | **P** |
| --- | --- | --- | --- | --- | --- | --- |
| Body mass index | -0.001 | 0.001 | 0.241 | 1 | 169 | 0.624 |
| Nestling age (in days) | 0.009 | 0.011 | 0.682 | 1 |  | 0.408 |
| Brood size | 0.020 | 0.010 | 3.798 | 1 |  | 0.051 |
| Sex | -0.031 | 0.020 | 2.364 | 1 |  | 0.124 |
| **Season (week number)** | **-0.023** | **0.011** | **4.704** | **1** |  | **0.030** |
| Time of the day (hour) | 0.017 | 0.013 | 2.747 | 1 |  | 0.097 |
| 450nm | -0.005 | 0.013 | 0.139 | 1 |  | 0.709 |
| **Intercept** | **0.377** | **0.015** | **652.489** | **1** |  | **<0.001** |

**Full model output bacteria-killing:**

Table S7 Model output of a linear mixed model exploring the bacteria-killing score (standardized per year) in relation to parental pair morph combination (either mixed- or like-morph). Covariates fitted in the model were nestling age (in weeks), brood size, sex (male as reference), season (in weeks) and time of the day. Random effect was territory ID. All continuous variables were scaled. Statistically significant variables indicated in bold. Sample size is 168.

| **Variable** | **Estimate** | **SE** | **χ2** | **ndf** | **ddf** | **P** |
| --- | --- | --- | --- | --- | --- | --- |
| Pair morph | 0.008 | 0.030 | 0.077 | 1 | 161 | 0.783 |
| Nestling age (in days) | 0.016 | 0.012 | 1.744 | 1 |  | 0.187 |
| Brood size | 0.015 | 0.013 | 1.486 | 1 |  | 0.225 |
| **Sex** | **-0.064** | **0.024** | **7.399** | **1** |  | **0.007** |
| Season (week number) | 0.002 | 0.014 | 0.032 | 1 |  | 0.858 |
| Time of the day (hour) | -0.001 | 0.012 | 0.006 | 1 |  | 0.939 |
| **Intercept** | **0.398** | **0.024** | **270.629** | **1** |  | **<0.001** |

Table S8 Model output of a linear mixed model exploring the bacteria-killing score (standardized per year) in relation to individual body condition. Covariates fitted in the model were nestling age (in weeks), brood size, sex (male as reference), season (in weeks) and time of the day (in hours). Random effect was territory ID. All continuous variables were scaled. Statistically significant variables indicated in bold. Sample size is 168.

| **Variable** | **Estimate** | **SE** | **χ2** | **ndf** | **ddf** | **P** |
| --- | --- | --- | --- | --- | --- | --- |
| Body mass index | 0.001 | 0.001 | 0.894 | 1 | 161 | 0.344 |
| Nestling age (in days) | 0.014 | 0.013 | 1.217 | 1 |  | 0.270 |
| Brood size | 0.017 | 0.013 | 1.714 | 1 |  | 0.190 |
| **Sex** | **-0.064** | **0.024** | **7.940** | **1** |  | **0.005** |
| Season (week number) | 0.004 | 0.014 | 0.070 | 1 |  | 0.791 |
| Time of the day (hour) | -0.002 | 0.012 | 0.028 | 1 |  | 0.866 |
| **Intercept** | **0.403** | **0.020** | **410.339** | **1** |  | **<0.001** |

**Full model output lipopolysaccharide challenge:**

Table S9 Model output of a linear model exploring the haptoglobin response in a lipopolysaccharide immune challenge in relation to parental pair morph combination (either mixed- or like-morph). Response variable was the delta value (post-LPS concentration subtracted by the baseline concentration). Covariates fitted in the model were nestling age (in weeks), brood size, sex (male as reference), season (in weeks), the baseline haptoglobin concentration and a reading at 450 nm of the baseline plasma sample to control for plasma redness. Random effect was territory ID but explained 0% variance. All continuous variables were scaled. Statistically significant variables indicated in bold. Sample size is 49.

| **Variable** | **Estimate** | **SE** | **χ2** | **ndf** | **ddf** | **P** |
| --- | --- | --- | --- | --- | --- | --- |
| Pair morph | -0.158 | 0.307 | 0.265 | 1 | 41 | 0.607 |
| Nestling age (in days) | 0.086 | 0.154 | 0.312 | 1 |  | 0.577 |
| Brood size | -0.007 | 0.163 | 0.002 | 1 |  | 0.968 |
| Sex | -0.494 | 0.310 | 2.542 | 1 |  | 0.111 |
| Season (week number) | -0.161 | 0.163 | 0.970 | 1 |  | 0.324 |
| Baseline haptoglobin conc. | -0.184 | 0.149 | 1.512 | 1 |  | 0.220 |
| 450 nm baseline | 0.047 | 0.146 | 0.103 | 1 |  | 0.750 |
| Intercept | 0.255 | 0.231 | 1.224 | 1 |  | 0.275 |

Table S10 Model output of a linear model exploring the haptoglobin response in a lipopolysaccharide immune challenge in relation to individual body condition. Response variable was the delta value (post-LPS concentration subtracted by the baseline concentration). Covariates fitted in the model were nestling age (in weeks), brood size, sex (male as reference), season (in weeks), the baseline haptoglobin concentration and a reading at 450 nm of the baseline plasma sample to control for plasma redness. Random effect was territory ID but explained 0% variance. All continuous variables were scaled. Statistically significant variables indicated in bold. Sample size is 49.

| **Variable** | **Estimate** | **SE** | **χ2** | **ndf** | **ddf** | **P** |
| --- | --- | --- | --- | --- | --- | --- |
| Body mass index | -0.001 | 0.007 | 0.021 | 1 | 41 | 0.884 |
| Nestling age (in days) | 0.138 | 0.152 | 0.820 | 1 |  | 0.365 |
| Brood size | 0.014 | 0.158 | 0.008 | 1 |  | 0.931 |
| Sex | -0.370 | 0.303 | 1.489 | 1 |  | 0.222 |
| Season (week number) | -0.133 | 0.160 | 0.700 | 1 |  | 0.403 |
| Baseline haptoglobin conc. | -0.179 | 0.154 | 1.354 | 1 |  | 0.244 |
| 450 nm baseline | -0.002 | 0.145 | 0.001 | 1 |  | 0.991 |
| Intercept | 0.195 | 0.209 | 0.870 | 1 |  | 0.351 |

**Body mass index residuals and body mass index percentiles used for survival analysis:**


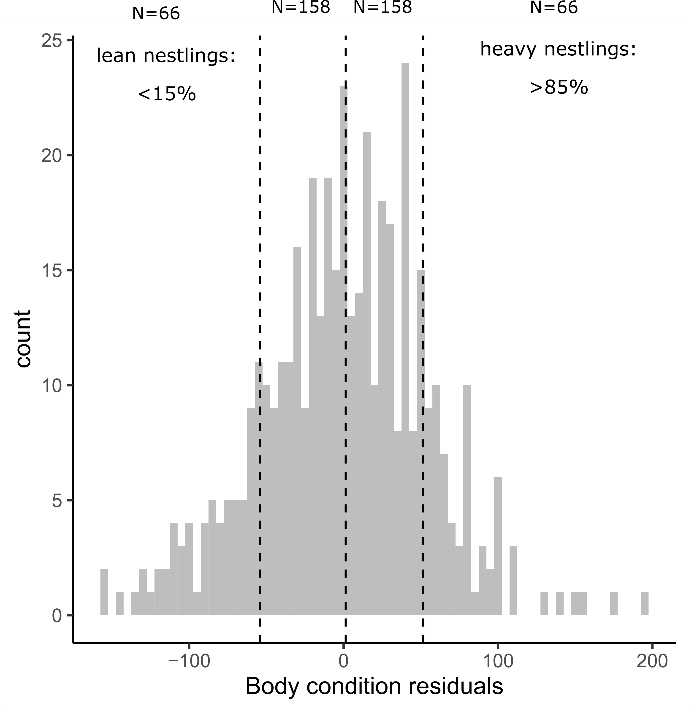


Figure S1 shows the histogram of the black sparrowhawk nestling body mass index residuals. Dashed vertical lines correspond, from left to right, to the 15% percentile boundary, the 0% boundary, 85% boundary of the body condition percentiles used for the MARK mark-recapture survival analysis. Individuals on the left size of the histogram are very lean (low body mass index) whereas chicks on the very right are heavy (high body mass index). The sample sizes for each body mass index percentile are depicted above the histogram.
